# Poly- and Perfluoroalkyl Substances (PFAS): Do They Matter to Aquatic Ecosystems?

**DOI:** 10.3390/toxics11060543

**Published:** 2023-06-19

**Authors:** Sipra Nayak, Gunanidhi Sahoo, Ipsita Iswari Das, Aman Kumar Mohanty, Rajesh Kumar, Lakshman Sahoo, Jitendra Kumar Sundaray

**Affiliations:** 1 Fish Genetics & Biotechnology Division, ICAR-Central Institute of Freshwater Aquaculture, Bhubaneswar 751002, Odisha, India; 2 Department of Zoology, Utkal University, Bhubaneswar 751004, Odisha, India; 3 Aquaculture Production and Environment Division, ICAR-Central Institute of Freshwater Aquaculture, Bhubaneswar 751002, Odisha, India

**Keywords:** perfluoroalkyl substances (PFASs), toxicants, bioaccumulation, fish, human health risks

## Abstract

Poly- and perfluoroalkyl substances (PFASs) are a group of anthropogenic chemicals with an aliphatic fluorinated carbon chain. Due to their durability, bioaccumulation potential, and negative impacts on living organisms, these compounds have drawn lots of attention across the world. The negative impacts of PFASs on aquatic ecosystems are becoming a major concern due to their widespread use in increasing concentrations and constant leakage into the aquatic environment. Furthermore, by acting as agonists or antagonists, PFASs may alter the bioaccumulation and toxicity of certain substances. In many species, particularly aquatic organisms, PFASs can stay in the body and induce a variety of negative consequences, such as reproductive toxicity, oxidative stress, metabolic disruption, immunological toxicity, developmental toxicity, cellular damage and necrosis. PFAS bioaccumulation plays a significant role and has an impact on the composition of the intestinal microbiota, which is influenced by the kind of diet and is directly related to the host’s well-being. PFASs also act as endocrine disruptor chemicals (EDCs) which can change the endocrine system and result in dysbiosis of gut microbes and other health repercussions. In silico investigation and analysis also shows that PFASs are incorporated into the maturing oocytes during vitellogenesis and are bound to vitellogenin and other yolk proteins. The present review reveals that aquatic species, especially fishes, are negatively affected by exposure to emerging PFASs. Additionally, the effects of PFAS pollution on aquatic ecosystems were investigated by evaluating a number of characteristics, including extracellular polymeric substances (EPSs) and chlorophyll content as well as the diversity of the microorganisms in the biofilms. Therefore, this review will provide crucial information on the possible adverse effects of PFASs on fish growth, reproduction, gut microbial dysbiosis, and its potential endocrine disruption. This information aims to help the researchers and academicians work and come up with possible remedial measures to protect aquatic ecosystems as future works need to be focus on techno-economic assessment, life cycle assessment, and multi criteria decision analysis systems that screen PFAS-containing samples. New innovative methods requires further development to reach detection at the permissible regulatory limits.

## 1. Introduction

### 1.1. What Is PFAS?

According to the Organisation for Economic Co-operation and Development [1], aliphatic fluorinated carbon chains are found in a class of thousands of anthropogenic compounds known as poly- and perfluoroalkyl substances (PFASs). Throughout the entirety of their manufacturing cycles, polyfluoroalkyl and perfluoroalkyl chemicals are discharged into the aquatic environment (i.e., during their production, along the supply chains, product use, and disposal of industrial and consumer products). While indirect emission sources are defined as emissions from the transformation of their precursors, direct emission sources of PFASs are characterised as emissions throughout their product cycle. Because of their durability, ability to bioaccumulate and potential for negative impacts on living organisms, PFASs have drawn attention from the general public on a worldwide scale. In silico research provides a computational platform to screen the activity of a potential molecule against a given target and select the molecules with the highest potential activity for further in vivo and in vitro studies. The computational analysis reveals that PFOA, which binds to Vitellogenin1 with a binding energy of −8.4 Kcal/mol, will possess a negative impact on the early gametogenesis of aquatic organisms. Some of the environmentally relevant groups of PFASs have been given in Table 1.

### 1.2. Uses of PFAS

Growing pollutants, such as PFASs, are very difficult to naturally breakdown. Since about a century ago, electrochemical fluorination and other telomerization methods have been used to create PFASs. This family of compounds is extremely useful for various applications due to its high chemical resistance, hydrophobicity, lipophobicity, heat resistance, and extremely low friction coefficient. Unfortunately, once released into the environment, these same beneficial characteristics turn them into “forever chemicals” [3]. The applications of PFASs are numerous and varied. Its several formulations have been used for a variety of purposes, including waterproofing, surfactants, repellent cookware coatings, and firefighting foams and fireproofing, among others [4]. PFASs’ physical and chemical characteristics, such as its high degree of thermal and chemical stability due to the strength of its carbon-fluorine (C-F) bonds and its capacity to lower surface tension, can be attributed to its significant commercial success and widespread application [5]. PFASs are also found in many common home products, such as cookware and food packaging, because of their hydrophobic and oleophobic qualities. The textile sector uses the most PFASs and its precursors, followed by the paper packaging industry and aftermarket consumer goods.

## 2. Impact of PFASs on Environment

**Figure 1:**
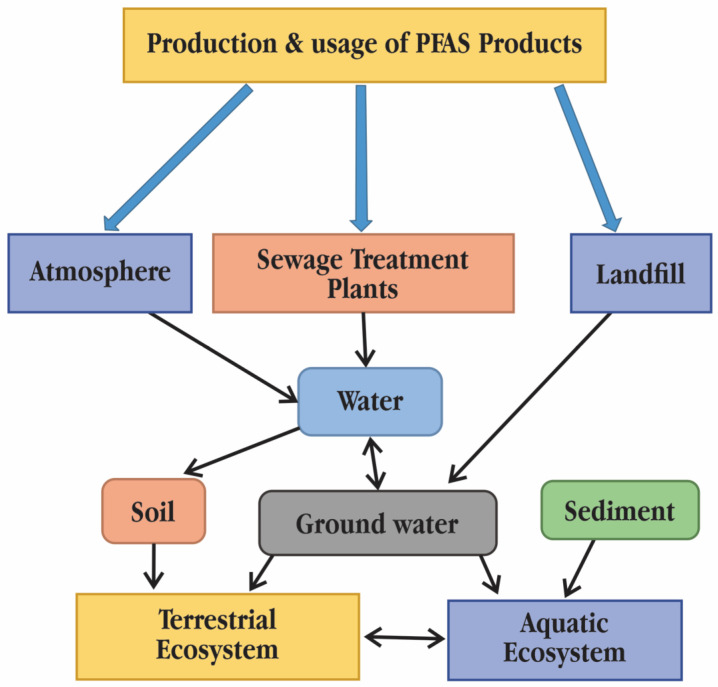
Pathways of polyfluoroalkyl and perfluoroalkyl substances (PFASs) into the environment and their fate [2]. © 2014 SETAC.

Traditional bioaccumulation measures have to be re-evaluated since PFASs and neutral lipophilic chemical compounds have very different bioaccumulation processes in animals. There is proof that phospholipids and proteins are crucial for the distribution and accumulation of PFAS in tissues [6]. Due to their persistence, propensity for bioaccumulation, and potential negative effects on living organisms, PFASs have drawn attention from the general public worldwide. Perfluorobutane sulfonic acid (PFBS) and perfluorobutanoic acid (PFBA), the two most prevalent short-chain PFASs, have been found in large quantities in drinking water, sediment, sewage sludge, and even snow and ice in the polar region. Leaching in landfills or dump sites, spraying of aqueous film forming foam (AFFF), and wastewater disposal and air emissions at manufacturing facilities are all ways that PFASs reach the environment (Figure 1). For a number of reasons, human exposure to PFASs is seen as ubiquitous. PFASs are known to bioaccumulate, accumulating in human and fish, in addition to being persistent and widespread. Because PFAS exposure has been linked to detrimental effects on human health, PFASs can be regarded as one of the most important groups of emerging contaminants or chemicals of emerging concern (CEC) [7]. Water is thought to be the primary means of PFASs’ transfer between environmental compartments and biota because of PFASs’ partial solubility in water, which significantly contributes to their diffusion. According to [8], wastewater treatment plants (WWTPs) frequently report high concentrations of perfluoroalkyl acids (PFAAs), which include both perfluoroalkyl carboxylic acids (PFCAs) and perfluoroalkane sulfonic acids (PFSAs). Although there are a few exceptions, such as bioconcentration factors (BCFs) and bioaccumulation factors (BAFs) (litres per kilogramme) of specific PFAAs in plankton, aquatic gill-ventilating invertebrates and fish generally rise with increasing perfluoroalkyl chain length and hydrophobicity [9]. Food webs of avian and marine mammals have the highest recorded trophic magnification factors (TMFs) for PFAA [10]. For instance, the TMF for perfluorooctane sulfonic acid (PFOS) in these rather lengthy food chains with fauna that breathes air (such as marine birds and other land animals) is around 20. TMFs are often substantially lower in aquatic piscivorous food webs. For instance, the TMFs of PFOS in the aquatic piscivorous food webs of Lake Ontario range from 1.9 to 5.9 [11]. This trend is consistent with earlier findings of low-octanol/water partition coefficients (KOW) and high octanol–air partition coefficient (KOA), moderately hydrophobic organic compounds, being biomagnified specifically for food webs [12]. All PFASs have the common feature of having extremely stable perfluorocarbon moieties in their molecular structure. Therefore, all PFASs either fully or partially change into very persistent PFASs in the environment and biota [13,14]. Perfluoroalkanes, one kind of PFAS, are thought to have been existed for thousands of years, according to studies. Therefore, even if environmental emissions stop right away, PFAS will persist in the environment for millennia or longer. Due to PFAS’s high persistence, they accumulate over time in the environment and in living organisms, which raises the potential for harm. When plants are cultivated on polluted soil or are irrigated with contaminated water, PFASs can accumulate in plants, including food crops. Through the food chains, bioaccumulation takes place, with apex predators (such as whales, bald eagles and humans) having the greatest amounts [10,15].

## 3. Impact of PFASs on Aquatic Ecosystem

**Figure 2:**
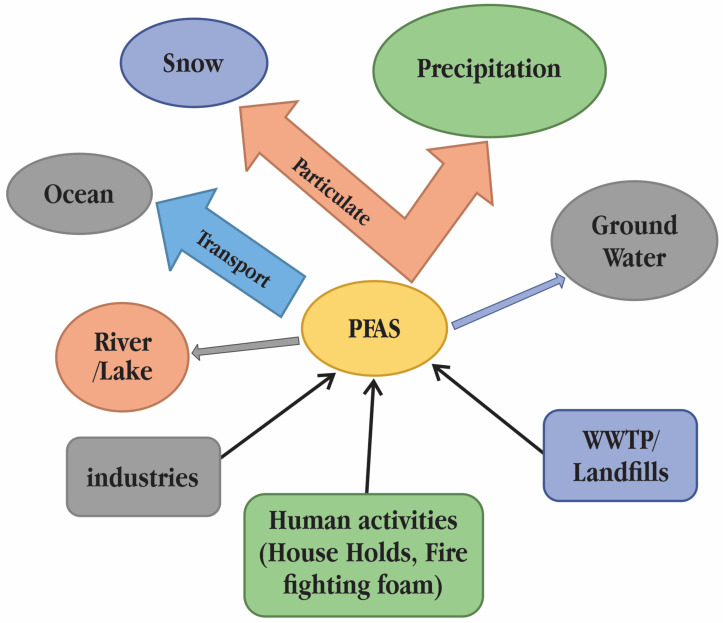
Sources of poly- and perfluoroalkyl substances (PFASs) contamination in surface water [18].

Long-chain PFASs and PFCAs have a high propensity to adhere to particles and have a large potential for bioaccumulation, whereas short-chain PFASs and PFCAs are usually found in the aqueous phase of the environment [16]. There are concerns about how the presence of PFASs in the aquatic environment may impact the flora and fauna in hydro systems (Figure 2). Physiologically based toxicokinetic (PBTK) models with absorption, distribution, metabolism, and excretion metrics have been created to assess the toxicokinetic of PFOS and perfluorooctanoic acid (PFOA) in various animal models, including fish and mammals [17]. These models are particularly useful for assessing the influence of membrane transporters and illuminating the considerable challenges in PFAS bioaccumulation equilibrium modelling.

### 3.1. Impact on Different Aquatic Organisms

**Figure 3:**
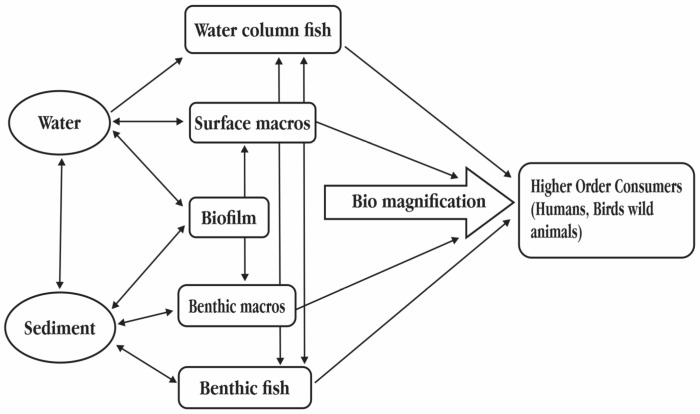
Poly- and perfluoroalkyl substances (PFASs) exposure pathways to aquatic environment.

Perfluoroalkyl acids (PFAAs) are a class of perfluorinated substances that have a carbon backbone that is completely fluorinated and are charged with functional hydrophilic groups, such as carboxylate, sulfonate, or phosphonate [19,20]. Surface runoff, product deterioration, or wastewater discharge are three ways through which PFAAs may enter the aquatic environment [21]. PFAS exposure pathways to aquatic environment has been given in Figure 3. Additionally, the bioaccumulation potential of PFASs differs across different species and individuals as well as depending on their physicochemical characteristics, such as their branched or linear chains, lengths of their chains, and functional groups. Additionally, it has been demonstrated that the PFAS structure affects the removal rate. For instance, linear isomers are removed more quickly than branching isomers [22]. Additionally, PFAS buildup and removal are influenced by the species, sex, and stage of pregnancy [23]. Numerous earlier investigations have shown that aquatic species experienced oxidative damage after being exposed to PFAAs. In *Oreocromis niloticus* cultured hepatocytes, exposure to 0–30 mg/L 127 PFOS and PFOA enhanced superoxide dismutase (SOD), catalase (CAT), and/or glutathione reductase activity, which resulted in reactive oxygen species (ROS). Additionally, a drop in glutathione (GSH) concentration and a rise in lipid peroxidation were observed [24]. Similarly, exposure to PFOS increased ROS generation in zebrafish (*Danio rerio*) embryos while also significantly increasing antioxidant activity [25]. Following PFOS exposure, adult *D. rerio* had enhanced ROS generation in the liver along with reduced CAT and elevated SOD activity [26]. Similar to adult fish, *D. rerio* larvae produced ROS after 24 h of exposure to perfluorononanoic acid (PFNA), and there were noticeable changes in the expression of the Sod1/2 and Gpx1 genes. It has been determined that a number of variables affect the toxicological effects of PFAAs on aquatic life. Sex affects the toxicity of PFAAs. In response to PFOA, *Gobiocypris rarus* displayed a sexual variation in the degree of protein expression. By being exposed to PFOS, male *D. rerio* developed a noticeable lipid buildup in their livers, whereas females showed a smaller accumulation. These findings suggest that sexual traits have an impact on PFAA-induced lipid metabolism. Male fish in mature *D. rerio* accumulated more PFNA than female fish did, and the transcription of genes associated with the reproductive system varied depending on sex, with levels of 17b-hsd, cyp19a, and star increasing in the female gonad while decreasing in the male gonad [27]. Additionally, PFOA exposure in male *Pimephales promelas* resulted in a considerable rise in thiobarbituric acid reactive substances (TBARSs) but not in females. *G. rarus* was exposed to PFOA, which raised the expression of methionine sulfoxide reductase B (MSRB) and peroxiredoxin (PRX) in both male and female fish. Only female *Oryzias latipes* exposed to PFOA had distinct expression patterns for osmolytes, such as trimethylamine N-oxide, whereas male fish had significantly higher levels of dimethylamine and myo-inositol. Life cycle exposure to *Oryzias melastigma*’s PFBS lowered the moist eye weight and increased the contents of choline, Gamma-aminobutyric acid (GABA), norepinephrine, and glutamate in female fish, but similar changes were not seen in male fish, indicating a sexual variation in ocular toxicity [28]. Consequently, one of the mechanisms causing variations in PFAA toxicity might be sexual differences. The biological toxicity of different chemical species of the PFAS family on different organisms has been given in Table 2.

#### 3.1.1. Impact on Fish

The expression of genes involved in lipid metabolism, energy generation, RNA processing, protein creation/degradation, and contaminant detoxification changed in largemouth bass exposed to high PFAS concentrations; these alterations are all in line with biomarker responses shown in other PFAS investigations. On average, PFOS levels were high enough in virtually all fish species to warrant recommendations against eating fish, but more research is required.

#### 3.1.2. Impact on Different Developmental Stages of Fish

**Figure 4:**
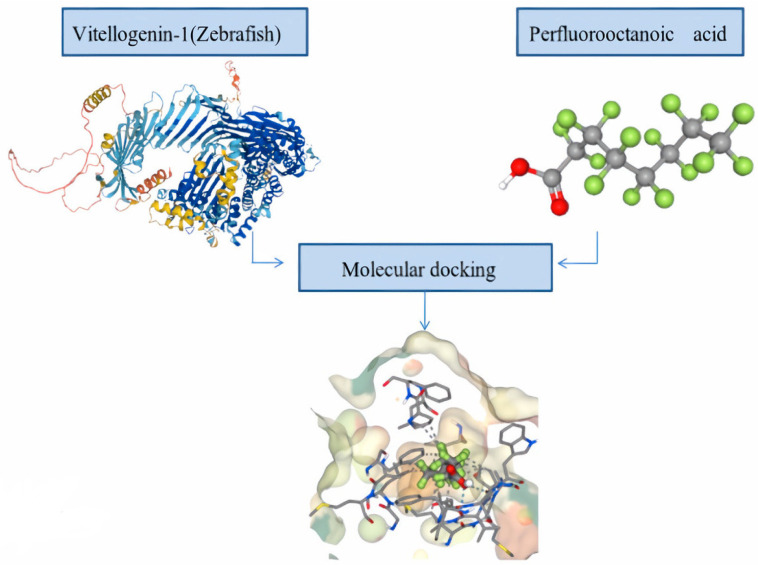
The docking pose of perfluorooctanoic acid (PFOA) with vitellogenin-1 (*D. rerio*) with binding affinity score of −8.4 kcal/mol. Arrow mark points towards the ligand–target interaction.

Acridine orange fluorescence demonstrated an increase in apoptosis in *Danio rerio* larvae exposed to PFOS. According to this research, PFOS exposure causes apoptosis, which is accompanied by a considerable shift in the expression of oxidative stress indicators [40]. There may be connections between several molecular pathways that cause PFAA-induced toxicity. There is a significant link between reproductive toxicity, oxidative stress, and developmental toxicity. Oxidative stress can influence egg hatching and larval deformity [29]. *O. latipes* embryos subjected to silver nanoparticles showed abnormalities along with a substantial shift in oxidative stress indicators, indicating that oxidative stress might have an impact on developmental processes [41]. PFAAs have an impact on how lipids, such as cholesterol, which is a precursor to sex hormones, are metabolised [42]. These findings imply that endocrine disturbances may be linked to PFAA-induced changes in lipid metabolism. Additionally, Gao et al. [43] demonstrated that Oestrogen receptors (ERs) regulate the genes for acyl-CoA oxidase 1 (Acox1) and pyruvate dehydrogenase kinase 4 (PDK4), which could inhibit the pyruvate dehydrogenase involved in the conversion of pyruvate to acetyl-CoA. This suggests that PFAA-induced endocrine disruption is connected to both carbohydrate and lipid metabolism. Neurotoxicity, followed by motor impairment and predator population decline, can result from thyroid disturbance, which is followed by developmental disruption, metabolic change, and apoptosis related to oxidative stress. Thyroid hormones, such as T3 and T4 in particular, caused fish to become more masculine, whereas thyroid hormone synthesis inhibitors, such as perchlorate, caused fish to become more feminine [44]. It seems that the reproductive system and thyroid disturbance are related. PFAAs inhibit larval development. The disturbance of metabolism and lipid metabolism, followed by a drop in adenosine triphosphate (ATP) content and developmental harm may be responsible for this outcome [29,45]. The main component of the yolk of vertebrate eggs is vitellogenin. As essential nutrients for developing embryos, all fishes manufacture vitellogenins. The majority of the components required to create and support a new life are delivered to the ooplasm of oocytes by vitellogenin when they develop by orders of magnitude during oogenesis. It is likely that PFASs are incorporated into the maturing oocytes during vitellogenesis and are bound to vitellogenin and other yolk proteins [46]. In the computer-aided drug designing process, protein–ligand docking is an important tool that predicts the interaction between the target and the ligand, both in terms of its structure, to find likely binding modes and energetics to estimate binding affinity. Here, we performed an in silico investigation analysis (molecular docking), to find out the potential binding interaction of perfluorooctanoic acid (PFOA) with vitellogenin-1, the egg precursor protein of *D. rerio.* The crystalline structure of vitellogenin-1 was extracted from AlphaFold (https://alphafold.ebi.ac.uk/ accessed on 9 November 2022)), an artificial intelligence (AI) system developed by Deep Mind in collaboration with the European Molecular Biology Laboratory’s European Bioinformatics Institute (EMBL-EBI) to predict the most accurate structure of proteins, from their amino acid sequence. The Protein Data Bank (PDB) structure of Vitellogenin-1 was validated using SAVES server (https://saves.mbi.ucla.edu/ (accessed on 9 November 2022). The three-dimensional structure of perfluorooctanoic acid was extracted from the Pubchem database (https://pubchem.ncbi.nlm.nih.gov/ (accessed on 9 November 2022). Molecular docking was performed using SeamDock server (https://bioserv.rpbs.univ-paris-diderot.fr/services/SeamDock/ accessed on 9 November 2022). SeamDock is an online user-friendly web server that integrates different docking tools in a common framework that makes it possible to undergo ligand global and/or local docking and a hierarchical approach combining the two for easy interaction site identification. For performing molecular docking, we have used the Autodock Vina platform in the SeamDock. The different parameters to perform docking (spacing, mode number, exhaustiveness, and energy range) were set and the docking program was launched. The Autodock docking output shows PFOS binds to vitellogenin-1 with a binding affinity of −8.4 kcal/mol (root-mean-square deviation (RMSD) value) (Figure 4). The interaction consists of a series of hydrophobic contacts and hydrogen bonds in the complex. This computational analysis indicates towards the fact that PFOA may have a negative impact on the reproductive cycle of the fish population as well.

#### 3.1.3. Impact on Different Organs of Fish

Since PFASs have a high affinity for serum albumin and fatty-acid-binding proteins, their distribution in the biota is tissue-dependent [47]. For example, in a variety of freshwater fish species from Beijing, China, the tissue distribution for PFASs changed from blood to liver and brain to muscle [2]. PFAA-induced oxidative stress has been recorded in aquatic species other than fish, despite the fact that there has only been limited research on fish. In *Oreocromis niloticus* cultured hepatocytes, exposure to 0–30 mg/L PFOS and PFOA increased superoxide dismutase (SOD), catalase (CAT), and/or glutathione reductase activity, which resulted in ROS. In addition, Liu et al. [24] found that glutathione content decreased, and lipid peroxidation increased. The nrf2, nqo1, and ho-1 genes were upregulated in the liver of the dark-spotted frog *Pelophylax nigromaculatus* (formerly *Rana nigromaculata*) in response to PFOA [48]. Together, these results imply that the Jun N-terminal kinase 1 (JNK1) and p38-dependent MAPK pathway may be the main player in the antioxidant activation brought on by PFAA treatment. According to [49], the glutathione S-transferase (GST) activity of *Daphnia magna* increased as a result of PFOS exposure. In *Paramecium caudatum*, exposure to 100 M PFOA considerably increased ROS production, while exposure to 10 M PFOA also increased ROS production [50]. In response to exposure to 2, 6, and 10 mg/L PFOS, *Unio ravoisieri* displayed enhanced SOD and CAT activities as well as elevated malondialdehyde (MDA) [51]. Perfluorododecanoic acid (PFDoA) treatment caused *Chlamydomonas reinhardtii* to produce more ROS and MDA. Following exposure to perfluorooctylphosphonic acid (PFOPA) for 6 or 24 h, an increase in the expression of ascorbate peroxidase I (apxI) transcription was seen [52]. It was also discovered that PFAA caused disruptions in lipid metabolism in fish tissues and the circulatory system. The serum LDL/VLDL levels and ATP content dropped, indicating hepatic lipid buildup in male fish. The liver total cholesterol and total glycerol content increased with increasing liver size [45]. *D. rerio* exposure to PFOS raised the liver’s total triglyceride/total cholesterol content while lowering serum triglyceride levels [53]. Hepatocyte viability in *Oreochromis niloticus* was decreased by PFOA and PFOS [54]. The survival of the *D. rerio* liver cells was reduced by PFOA, PFBA, and Perfluorohexanoic acid (PFHxA), and PFBA caused the development of lipid droplets, suggesting PFAA hepatotoxicity. After exposure to PFOS, the liver of male Zebrafish displayed lipid droplet buildup [38]. *Danio rerio* treated with perfluoro-N-decanoic acid (PFDA) had a greater triglyceride to total cholesterol ratio (TG/TC) in the liver and a lower ratio in the serum. *Danio rerio* showed an increase in hepatocyte vacuolization and lipid droplet number in response to PFOS [55]. Additionally, when compared to the control, fish exposed to either PFOS or PFOA significantly increased their hepatosomatic index (HSI) [55]. In *D. rerio*, PFOS exposure decreased serum TC and TG levels while elevating liver TG and TC levels with rising HIS as demonstrated by Cheng et al. [55]. According to some research, the formation of PFAS-relevant cellular and molecular targets may be connected, at least in part, to the compound’s rising toxicity with exposure time. As known hepatotoxins, PFASs have been well-documented targets of the liver’s hepatocytes and the enzymes involved in phase I hepatic detoxification, particularly cytochrome P450 [56]. Recent studies in zebrafish have shown that the differentiation of the liver and hepatic enzymes may be responsible for the stage-dependent increase in the embryotoxicity of both PFOA and perfluoroether carboxylic acids (PFECAs) as well as other hepatotoxins [1,57,58]. Xu et al., found that *Coryphaena hippurus* (mahi-mahi) express genes involved in hepatocyte differentiation and related liver enzymes during a time window (i.e., 36–48 h post fertilization (hpf)) that corresponds with increased embryotoxicity in the current study, suggesting that hepatic development may also play a part in this toxicity [59]. It is unclear whether the observed enhanced toxicity is brought on by the chorion barrier breach, liver growth, potentially other targets, or just the prolonged duration of the exposure (and cumulative effects).

### 3.2. Biofilm as a Bio-Indcator of PFAS Contamination

In aquatic environments, biofilm is one of the most widely dispersed producers [60]. It has been used as a natural sampling tool to track the spatial variability of anthropogenic pollutants, such as heavy metals (copper, zinc, and cadmium) and hydrophobic organic pollutants, such as polychlorinated biphenyls [61], pesticides, and herbicides. Copper, arsenic, and cadmium were the main sources of heavy metal contamination in oysters from the northern South China Sea, followed by lead and mercury [62]. Copper (Cu) stable isotopes in transplanted oysters can therefore act as a new tool for monitoring anthropogenic metal bioaccumulation in marine environments, as demonstrated by [63]. They show that Cu isotopes can constrain the continental Cu fraction bioaccumulated in Pacific oysters (*Crassostrea gigas*) and infer its natural or anthropogenic origin. Mussels (*Mytilus edulis*) can bioaccumulate Lithium (Li) in their soft tissues, which suggests that they could be used as biomonitoring organisms for Li pollution in coastal water, according to research by [64]. However, little is understood about how biofilm affects PFAS fate in aquatic systems [65,66]. Additionally, it is common practice to evaluate the negative effects of hazardous chemicals on aquatic ecosystems using the microbial diversity and sensitivity of biofilms [67]. Given that PFASs are common in aquatic settings, biofilms should be exposed to high concentrations of PFASs, yet their interactions have not yet been comprehensively investigated. Low levels of the fungal community’s alpha diversity in biofilms were similarly correlated with high PFAS levels. A useful biomarker for identifying the presence of PFASs in aquatic habitats is biofilm. In Taihu Lake, China, where prior research suggested that PFAS contamination is widespread, surface water, biofilm, phytoplankton, and freshwater snails (*Cipangopaludina chinensis*, Gastropoda) were collected [68]. To further comprehend the significance of biofilm in PFAS transfer in the environment, the trophic transfer of PFASs from biofilm to snails was assessed. Additionally, the effects of PFAS pollution on aquatic ecosystems were investigated by evaluating a number of characteristics, including extracellular polymeric substances (EPSs) and chlorophyll content as well as the diversity of the microorganisms in the biofilms Zhang et al. [69] claim that biofilms can efficiently acquire PFASs and can accurately describe the levels, profiles, and geographical trend of PFASs in a body of water. They first reported the presence of chlorinated polyfluoroalkyl ether sulfonic acid (Cl-PFESAs, trade name F-53B) in periphytic biofilm. The study found that the colonised biofilms successfully bioaccumulated PFASs from water, with the total concentration (PFASs) ranging from 1.96–20.1 ng/g wet weight, and that the bioaccumulation factor grew with the PFASs log Kow values. The PFASs in biofilms showed a greater association with those in water compared to phytoplankton. The PFAS concentrations in biofilm are likely to be more representative than in grab samples, making it a suitable integrative sample.

## 4. Effect of PFAS on Gut Microbiome of Aquatic Organisms

**Figure 5:**
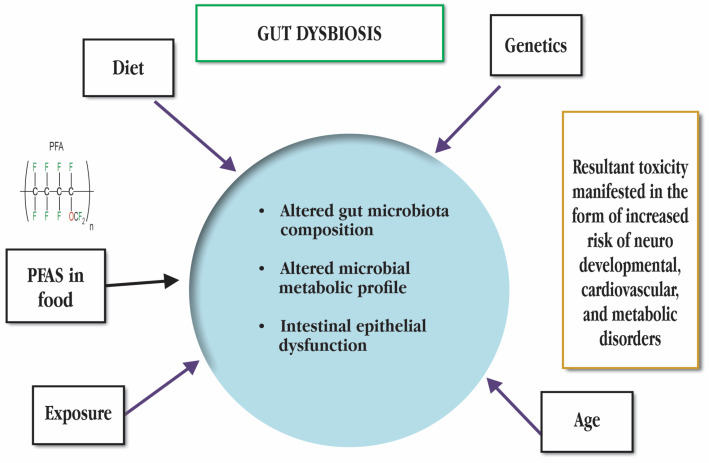
Factors responsible for gut dysbiosis. PFAS stands for Poly- and perfluoroalkyl substances.

Little is known about the effects of PFASs on aquatic species, such as fish, particularly at the molecular level of host gut–microbiome interactions. There are still some works in the higher levels, such as reptiles (turtles). One of the numerous perfluoroalkyl compounds (PFASs) utilised to increase product stain, grease, and water resistance is PFOS (perfluorooctane sulfonate). These substances are widely present in both food products and a variety of industrial products. PFASs are known to bioaccumulate, impair human and environmental health, and are highly persistent and common in aquatic habitats. Consequently, PFAS bioaccumulation has been documented in a number of freshwater and marine aquatic organisms [70,71]. It has been reported by [72] that PFOS increases yolk sack area in zebrafish larvae. The gut microbiota of freshwater turtles was also affected by PFAS exposure in terms of cysteine and methionine metabolism, sulphur metabolism, toluene degradation, and tyrosine metabolism. Both the gut microbiota of the turtle (host) and several pathways related to carbohydrates, energy, amino acids, nucleotides, co-factors, vitamins, terpenoids, and polyketides were disturbed [73]. Some of the factors responsible for gut dysbiosis are given in Figure 5. PFOS (perfluorooctane sulfonate) and PFOA (perfluorooctanoic acid) are persistent organic pollutants due to their long carbon chains and high-energy fluorocarbon bonds, which make them extremely stable [9]. As a result, some commercial alternatives for short carbon chains or semi-fluorinated analogues have evolved [74]. One of the novel substitutes for PFASs is sodium-perfluorous nonenoxybenzene sulfonate (OBS). It is widely employed in the fire protection industry, oil extraction, cleaning steel plates, printing, electroplating, and other industrial fields due to its reduced production costs and substantially greater cost performance. Environmental contamination issues undoubtedly followed as a consequence. The maximum concentration of OBS even reached 3.2 × 10^3^ ng/L in a lake close to the Daqing oil field, where it had been discovered [59]. Bao et al., found that OBS causes gut microbiota dysbiosis in adult zebrafish and is hardly biodegradable [75].

## 5. Impact of PFASs on Aquaculture

Previous research revealed that various fish species displayed varying quantities of PFASs even from the same fishing location, which may affect the levels of PFASs in the raw materials used to make fish meals and feeds. In addition to fish species, the fishing location has an impact on the presence and concentrations of PFASs in raw materials. PFOSs have often been detected at greater concentrations in fish than PFOA. Fish from regions with PFASs point to sources that have been reported to have increased levels of PFOA. If present in high amounts, drugs and pesticides with low atomic fractions of fluorine might cause water to have a moderate or high organofluorine content. During conventional sample extraction, other PFAS classes could be left out of the study. For instance, only 1:1 hexane/acetone was effective in removing nonpolar PFASs, such as perfluorobutyl side chain-fluorinated copolymer surfactant with molecular weight > 1600 g/mol, from soil, wastewater sludge, and sediment; methanol or acetonitrile yielded substantially lower recoveries [76]. While WWTPs discharge wastewater that is high in PFASs, which further contaminates rivers, lakes, and agricultural areas, e-waste contains PFASs that leak out into the groundwater, soil, and air. Additionally, PFASs absorption in plants, animals, and soil biota leads to ecotoxicity, and biosolids generated during the treatment process at WWTPs are frequently employed in agricultural areas as soil supplements [77].

## 6. Effect of PFASs on Humans

**Figure 6:**
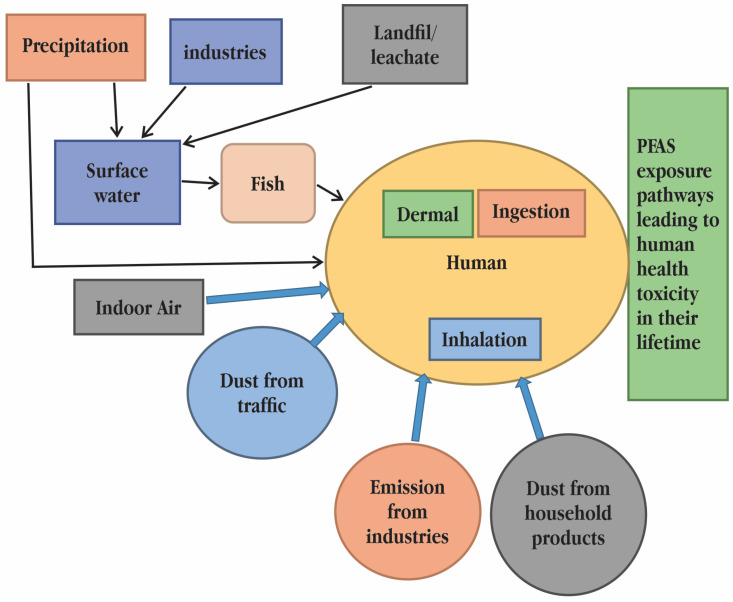
Poly- and perfluoroalkyl substances (PFAS) exposure pathways and their toxicological effects on human health [18].

Although air and air-suspended dust, food packaging, and cookware all contribute to the human body’s overall PFAS burden, water and food intake are typically regarded as the two main sources of PFASs in humans [78]. When there are high ambient concentrations of PFASs, humans can become exposed by consuming contaminated food and drink, agricultural goods, or game and fish. At polluted locations, PFAS environmental concentrations and exposures to humans and wildlife are often greatest. PFAS exposure can happen in a number of ways, but the severity of the exposure is influenced by the distance from the exposure source, the concentration of the source, and the frequency of exposure. Numerous investigations have shown that PFASs are present in samples of human blood as well as samples of aquatic and terrestrial flora and fauna. Because exposure to PFAS in low quantities can still have negative impacts on human health, it is crucial to find them as soon as possible. Some PFAS can induce reproductive and developmental abnormalities even at low quantities, according to epidemiological research [79]. Understanding PFAS exposure in humans requires careful assessment of PFASs in consumer items. Some investigations on particle-induced gamma-ray emission spectrometry (PIGE), X-ray photoelectron spectroscopy (XPS), and instrumental neutron activation analysis (INAA) have examined the fluorine concentration at the material surface or subsurface, while others take into account the average concentrations across the sample under study [80]. Understanding the total fluorine analysis in human tissues is made possible by the unknown organofluorine content. Numerous studies have demonstrated that behavioural and dietary heterogeneity accounts for the difference in PFAS exposure between children and adults. Based on the different composition of PFASs detected in blood, research from the Faroe Islands revealed that hand-to-mouth contact with automobile petting was a significant exposure source for children but not for adults. PFAS exposure pathways and their toxicological effects on human health has been given in Figure 6. In areas where it had previously been hidden, toxicity is now obvious because of evolving regulatory requirements. The Environmental Protection Agency (EPA) ultimately released a health alert for PFOA and PFOS in drinking water in 2016 at a concentration of 0.07 ppb (or 70 ppt) [81]. Many toxicologists and environmentalists have pushed for a 1 ppt safety threshold for PFOA and PFOS in recent years. Moreover, many states in the United States have proposed exposure levels that are significantly lower than the EPA’s existing guidelines after testing for dozens of additional PFAS [82]. The amount of PFASs detected in human plasma and serum varies depending on the population, ranging from single- or double-digit micrograms per litre in the general population to hundreds or even thousands of micrograms per litre in occupationally exposed workers and humans living close to contaminated sites [83]. Geographical location, PFAS type, sex, and age also affect observed concentrations. Due to the widespread usage of PFASs in several consumer items, exposure to humans and wildlife has occurred as a result of environmental discharges. PFASs were formerly thought to be biologically inert, but over time it was learned that they might have hazardous effects on organisms. They have extremely long elimination half-lives (3–5 years) in humans and animals due to their unusual binding mechanisms in biological tissues, which are a result of their oleophobic nature (they bind to proteins instead of adipose tissue), and this gives them plenty of time to accumulate in concentrations in humans and wildlife and potentially cause negative side effects [84]. PFASs can enter the umbilical cord blood during pregnancy through the placenta of the mother, albeit various PFASs have varying degrees of ability to do so. For example, formula made with contaminated water, breast milk from PFAS-exposed mothers, and the more common hand-to-mouth actions of young children when crawling and playing on the floor can all result in toddlers and babies ingesting PFASs [85].

## 7. Potential Molecular Mechanism of PFASs

The primary mechanism of hepatotoxicity, according to the study’s authors, is the disruption of the expression of genes regulating lipid homeostasis. The outcomes obtained by Rosen et al. [86] may lend weight to that view. The study showed that short-chain PFASs, such as PFNA and PFHxS, activated the peroxisome proliferator-activated receptors (PPARs) transcription factor receptor, increasing apolipoprotein I (apo A1) levels and fatty acid metabolism. Additionally, a decrease in lipoprotein lipase (LPL) and an increase in the high-density lipoprotein (HDL) fraction result in lower serum triglyceride levels and disturbances in sterol transport. Additionally, it was shown that, in comparison to PFOA, PFOS, and PFNA, the short-chain perfluorohexanesulfonic acid (*PFHxS*) induced an almost 10-fold greater expression of oxidoreductase, one of the regulators of lipid metabolism, and stearoyl coenzyme A desaturase (Scd) [87]. Additionally, it was demonstrated that PFBS induced adipogenesis in 3T3-L1 cells via activating the pathway mediated by extracellular signal-regulated kinases MAPK/ERK (mitogen-activated protein kinases). It appears that the process of inducing adipokinesis begins with receptors on the surface of cells, from which the signal is sent to the nucleus.

## 8. Conclusions and Future Perspectives

In the current review, we have outlined the effects of PFASs on the entire ecosystem, with a focus on the aquatic environment. We have also discussed how toxicity is understood, experienced, and imagined; the factors influencing regulatory action and ignorance; and how PFASs have been the focus of competing forms of knowledge production. Lack of efficient chemical control shifts the burden of demonstrating harm to exposed individuals, causing popular epidemiology cycles in which people organize and try to link their health issues to hazardous exposure [88]. Toxicity is frequently the result of the spatially scattered and chronologically accumulating “attritional catastrophes”, which describe a sort of harm that develops gradually over time. The impact of PFASs in humans has been outlined by many authors. At present, little is known about the environmental exposure, transport, and fate of these compounds, particularly PFOA in aquatic organisms. Based on current understanding of the developmental effects of PFOS and PFOA in aquatic organisms, several avenues of research are suggested that would further support the risk assessment of these perfluorinated organic chemicals.

The availability of analytical methods and instrumentation that can rapidly assess PFAS exposure in the field is limited. Research on techno-economic assessment, life cycle assessment, and multi criteria decision analysis systems for screening PFAS-containing samples is one of the priority area, and new innovative methods requires further development to reach detection at the regulatory permissible limits. Mission mode international collaboration to find out the pathways of widespread aquatic organism exposure in transboundary water bodies, which will reduce the exposure to humans via the food chain. Pharmacokinetic characterization of perfluoroalkyl compounds, especially during early stages of gametogenesis, reproduction and embryology in aquatic organisms, will greatly facilitate our understanding of chemical disposition and potential cellular targets. Cellular and molecular mechanistic findings of developmental toxicity will be instrumental in extrapolating the health risk potential of these compounds for aquatic organisms and humans. Research on public policies aimed at reducing PFAS exposure that could contribute to reductions in impact on primary and secondary food chain in aquatic ecosystem.

## Figures and Tables

**Table 1:** Environmentally relevant groups of polyfluoroalkyl and perfluoroalkyl substances (PFASs) in aquatic environments [2].

Compound Groups	Acronym	Formula	Chemical Structure	Typical PFASs
Perfluoroalkyl substances Perfluoroalkyl sulfonates	PFASs	CnF_2n+1_SO_3_^−^-	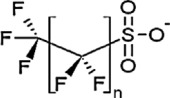	n = 3–9
Perfluoroalkyl carboxylates	PFCAs	CnF2_n+1_COO-	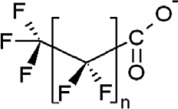	n = 1–17
Perfluoroalkyl phosphonates	PFPAs	CnF_2+1_(O)P(OH)O-	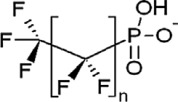	n = 4, 6, 8
Perfluoroalkyl sulfonamides	FASAs	CnF2_n+1_SO_2_NH_2_	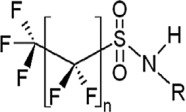	n = 8, R = H n = 8, R = CH_3_ n = 8, R = C_2_H_5_ n = 4, R = CH_3_
Perfluoroalkyl sulfonamidoethanols	FASEs	CnF_2n+1_SO_2_NHCH_2_CH_2_OH	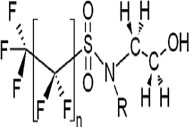	n = 8, R = H n = 8, R = CH_3_ n = 8, R = C_2_H_5_
Perfluoroalkyl sulfonamidoacetic acids	FASAAs	CnF_2n+1_SO_2_NHCH_2_COOH	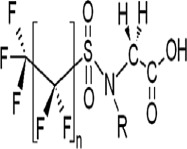	n = 8, R = H n = 8, R = CH_3_ n = 8, R = C_2_H_5_
Polyfluoroalkyl substances Polyfluoroalkyl phosphoric acid esters	PAPs	(O)P(OH)_3–x_ (OCH_2_CH_2_C_n_F_2n+1_)_x_	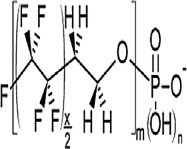	m = 1, n = 2, x:2 monoPAP m = 2, n = 1, x:2 diPAP
n:2 Fluorotelomer alcohols	n:2 FTOHs	C_n_F_2n+1_CH_2_CH_2_OH	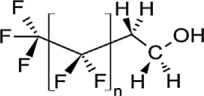	n = 4, 6, 8, 10
n:2 Fluorotelomer unsaturated aldehydes	n:2 FTUALs	C_n−1_F_2n−1_CF=HCHO	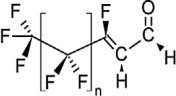	n = 3, 5, 7, 9
n:2 Fluorotelomer saturated aldehydes	n:2 FTALs	C_n_F_2n+1_CH_2_CHO	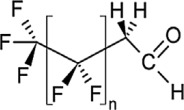	n = 4, 6, 8, 10

© 2014 SETAC.

**Table 2:** Adverse effects of perfluoroalkyl acids on different organisms [29].

Type of PFAS	Organisms (Species)	Impact	Reference
PFOS	*Danio rerio* embryos	Caused a delay in hatching; decrease in the hatching rate, larval survival rate, and body length; and developmental abnormality (yolk sac oedema).	[30]
	Daphnia magna	Number of days to the first brood increased, decreased first brood quantity, and decreased population’s intrinsic rate of growth.	[31]
	Oryzias melastigma	Larval malformation, such as bent spine, cardiac 710 oedema, and abdominal oedema growth.	[32]
PFOA	Freshwater microalgae *Chlamydomonas. reinhardtii* *Brachionus calyciflorus* Rare minnows (*Gobiocypris* *rarus*)	Significant inhibition of and decrease in the reproduction and intrinsic rate of natural increase, as well as an increase in generation time. Hypertrophy in liver, eosinophilic hyaline droplets in the hepatocytes cytoplasm, and eosinophilic hyaline droplets in hepatocytes.	[33,34]
PFDoA	*D. rerio* embryos/larvae	Decrease in acetylcholine (ACh) content and activity, with a decrease in swimming speed.	[35]
PFBS	*O. melastigma*	Decreased the eye wet weight, and increased the choline, GABA, norepinephrine, and glutamate contents in female fish.	[36]
PFAA	Cells of *Salmo salar*, *O. niloticus*, and *G. rarus*.	Changes such as increased fatty acid synthase transcription, reduced GSH, increased CAT activity, increased Casp3/8/9activity induction, CYP1A/3A transcription increase, Vtg protein content increase, and nuclear receptor activation.	[24]
PFNA	Zebrafish (*D. rerio*)	Pericardial oedema, spine crooked malformation, developmental delays.	[37]
	Zebrafish (*D. rerio*)	Enlarged follicles, hypertrophy of follicular epithelium, hyperplasia of follicle cell.	[38]
PFAA mixture (PFOA, PFOS, PFBS, PFNA)	Japanese medaka (*Oryzias latipes*)	Low fecundity, low hatching rate, and low larval survival rate	[39]

## Data Availability

Created or analyzed in this study.

## Abbreviations

**Acronym**	**Definition**
ACh	Acetylcholine
Acox1	Acyl-CoA oxidase 1
AFFF	Aqueous film forming foam
ATP	Adenosine triphosphate
ATSDR	Agency for Toxic Substances and Disease Registry
BAFs	Bioaccumulation factors
BCFs	Bioconcentration factors
CAT	Catalase
CECs	Chemicals of emerging concern
EPA	Environmental Protection Agency
ER	Estrogen receptor
GABA	Gamma-aminobutyric acid
GST	Glutathione S-transferase
HSI	Hepatosomatic index
INAA	Instrumental neutron activation analysis
ITRC	Interstate Technology and Regulatory Council
JNK1	Jun N-terminal kinase 1
KOA	Octanol-air partition coefficient
KOW	Octanol/water partition coefficients
LDL/VLDL	Low/Very-low-density lipoprotein
LPL	Lipoprotein lipase
MDA	Malondialdehyde
MSRB	Methionine sulfoxide reductase B
OBS	Oxybenzene sulfonate
PBTK	Physiologically based toxicokinetic
PDB	Protein Data Bank
PDK4	Pyruvate dehydrogenase kinase 4
PFAAs	Perfluoroalkyl acids
PFAS	Poly- and Perfluoroalkyl substances
PFBA	Perfluorobutanoic acid
PFBS	Perfluorobutane sulfonic acid
PFCAs	Perfluoroalkyl carboxylic acids
PFDA	Perfluoro-N-decanoic acid
PFDoA	Perfluorododecanoic acid
PFECA	Perfluoroether carboxylic acid
PFHXA	Perfluorohexanoic acid
PFNA	Perfluorononanoic acid
PFOA	Perfluorooctanoic acid
PFOPA	Perfluorooctylphosphonic acid
PFOS	Perfluorooctane sulfonate
PFSAs	Perfluoroalkane sulfonic acids
PIGE	Particle-induced gamma-ray emission spectrometry
PPARs	Peroxisome proliferator-activated receptors
PRX	Peroxiredoxin
RMSD	Root-mean-square deviation
ROS	Reactive oxygen species
SOD	Superoxide dismutase
TBARSs	Thiobarbituric acid reactive substances
TC	Total cholesterol
TG	Triglycerides
TMF	Trophic magnification factors
USEPA	United States Environmental Protection Agency
WWTPs	Wastewater treatment plants
XPS	X-ray photoelectron spectroscopy
